# Synthesis of COF-SO_3_H immobilized on manganese ferrite nanoparticles as an efficient nanocomposite in the preparation of spirooxindoles

**DOI:** 10.1038/s41598-023-49628-7

**Published:** 2023-12-20

**Authors:** Samira Moein Najafabadi, Javad Safaei Ghomi

**Affiliations:** Department of Organic Chemistry, Faculty of Chemistry, University of Kashan, Kashan, Islamic Republic of Iran

**Keywords:** Organic chemistry, Heterogeneous catalysis

## Abstract

The synthesis of sulfonamide-functionalized magnetic porous nanocomposites is highly significant in chemistry due to their exceptional properties and potential as catalysts. COFs are a new class of organic porous polymers and have significant advantages such as low density, high chemical and thermal stability, and mechanical strength. Therefore, we decided to synthesize COFs based on magnetic nanoparticles, by doing so, we can also prevent the agglomeration of MnFe_2_O_4_. MnFe_2_O_4_@COF–SO_3_H possesses a large specific surface area, supermagnetism, and is acidic, making it an optimal catalyst for organic reactions. This particular catalyst was effectively employed in the green and rapid synthesis of various spiro-pyrano chromenes, while several analytical techniques were utilized to analyze its structural integrity and functional groups. The role of a specific site of MnFe_2_O_4_@COF–SO_3_H was confirmed through different control experiments in a one-pot reaction mechanism. It was determined that MnFe_2_O_4_@COF–SO_3_H acts as a bifunctional acid–base catalyst in the one-pot preparation of spirooxindole derivatives. The formation of a spiro skeleton in the multicomponent reaction involved the construction of three new σ bonds (one C–O bond and two C–C bonds) within a single process. The efficiency of the MnFe_2_O_4_@COF–SO_3_H complex is investigated in the synthesis of spirooxindoles of malononitrile, and various isatins with 1,3‐dicarbonyles. The nanocatalyst demonstrated excellent catalytic activity that gave the corresponding coupling products good to excellent yields. Furthermore, the heterogeneous magnetic nanocatalyst used in this study demonstrated recoverability after five cycles with minimal loss of activity.

## Introduction

**Figure 1:**
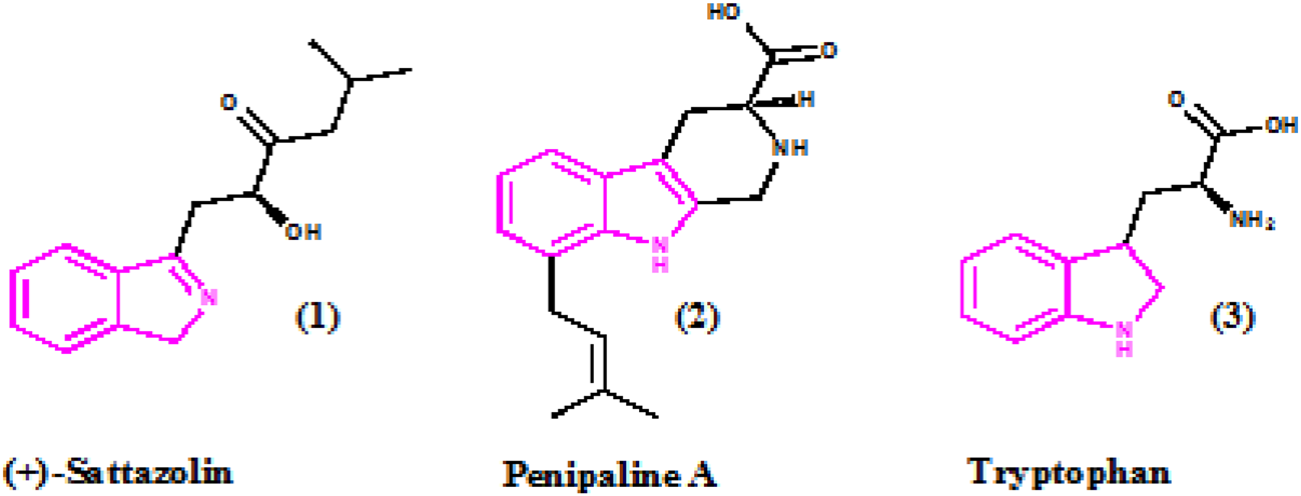
Indole scaffold in physiologically and pharmacologically relevant substances.

The indole scaffold is a highly important structure in the field of heterocyclic chemistry, which has a wide range of pharmacological and bioavailability usages, including its effectiveness as antibacterial, anti-diabetic, anti-hypertensive, anti-inflammatory, and anti-cancer properties (Fig. 1)^1–5^. As specified, compound (1) is a medication used for the treatment of headaches, compound (2) is a type of antibiotic medication used to treat bacterial infections such as pneumonia, bronchitis, and skin infections, compound (3) is an amino acid needed for normal growth in infants and for the production and maintenance of the body's proteins, muscles, enzymes, and neurotransmitters. It is an essential amino acid. This means your body cannot produce it, so you must get it from your diet. Moreover, it is evident that indolin-2-one and its derivatives have tremendous potential as anticancer drugs^6^.

In the academic context, it can be stated that the pyran structure is composed of a six-membered ring that contains a single heteroatom of oxygen, sulfur, selenium, or tellurium. This heteroatom is bonded to the cyclic system, which consists of two double bonds and one tetrahedral atomic center. Pyrans are very rare as natural products. Plants have been found to yield only a limited number of 2H-pyrans^7^.

In the last 10 years, the synthesis of spiro compounds has attracted substantial attention due to their extensive biological and medicinal applications. Isatins, also known as 1H-indole-2,3-diones, serve as a crucial foundation for the synthesis of various spiroheterocycles with both natural and synthetic origins^8,9^. These spiro heterocycles have shown diverse biological activities^10–12^.

Various techniques have been studied for the synthesis of oxospiro[indolin-pyrans] compounds^13^. However, these methods suffer from drawbacks such as long reaction times, elevated reaction temperatures, laborious workup, low yields of the products, and costly and non-reusable catalysts. Therefore, developing an environmentally friendly and straightforward synthetic approach for the production of oxospiro[indolin-pyrans] is highly significant.

Covalent organic frameworks (COFs) are a newly discovered category of porous polymers that exhibit strong covalent interactions between organic building blocks to form periodic skeletons^14–17^. COFs are advantageous due to their low density, enhanced chemical and thermal stability, remarkable mechanical strength, and high surface area^18^. In 2005^19^, the first examples of COF were reported by Yaghi and his team, and since then, they have found diverse applications in fields such as electronics^20^, storage and gas separation^21,22^, energy storage^23^, and catalysis^24^.

Additionally, nanoscale heterogeneous catalysts, acting as mediators between homogeneous and heterogeneous catalysts, can be conveniently dispersed in the reaction mixture compared to the bulk materials. Nevertheless, there is limited research available on reducing the size of COFs to the nanometer range to utilize them as active sites for catalysis^25,26^.

Magnetic nanoparticles (MNPs) are gaining attention as sustainable nanocatalysts for chemical transformations due to their effective activity, low cost, straightforward preparation method, high stability, and controlled separation by an external magnetic field^27,28^. Among the nanostructured magnetic materials, bimetallic oxide manganese ferrite (MnFe_2_O_4_) nanoparticles have unique physical and chemical properties, making them attractive for use in various applications such as sensors, filters, transformers, magnetic recordings, biomedical applications, catalysis, and supercapacitors^29,30^. MnFe_2_O_4_ is preferred over other ferrites due to its higher magnetic sensitivity and low resistance^31^.

The one-pot multicomponent reaction protocol is an efficient method for synthesizing nitrogen-containing heterocyclic compounds with significant applications in medicinal chemistry and organic synthesis^32^. Spirooxindole derivatives, which are significant fused heterocycles, serve as the fundamental building blocks of synthetic compounds, drug molecules, and natural products. While several techniques have been researched for the production of spiro 4H-pyrans, each procedure offers several benefits and disadvantages^33,34^. Therefore, it is essential to offer a fresh, effective method for creating these fused heterocyclic structures^35^.

**Scheme 1.**
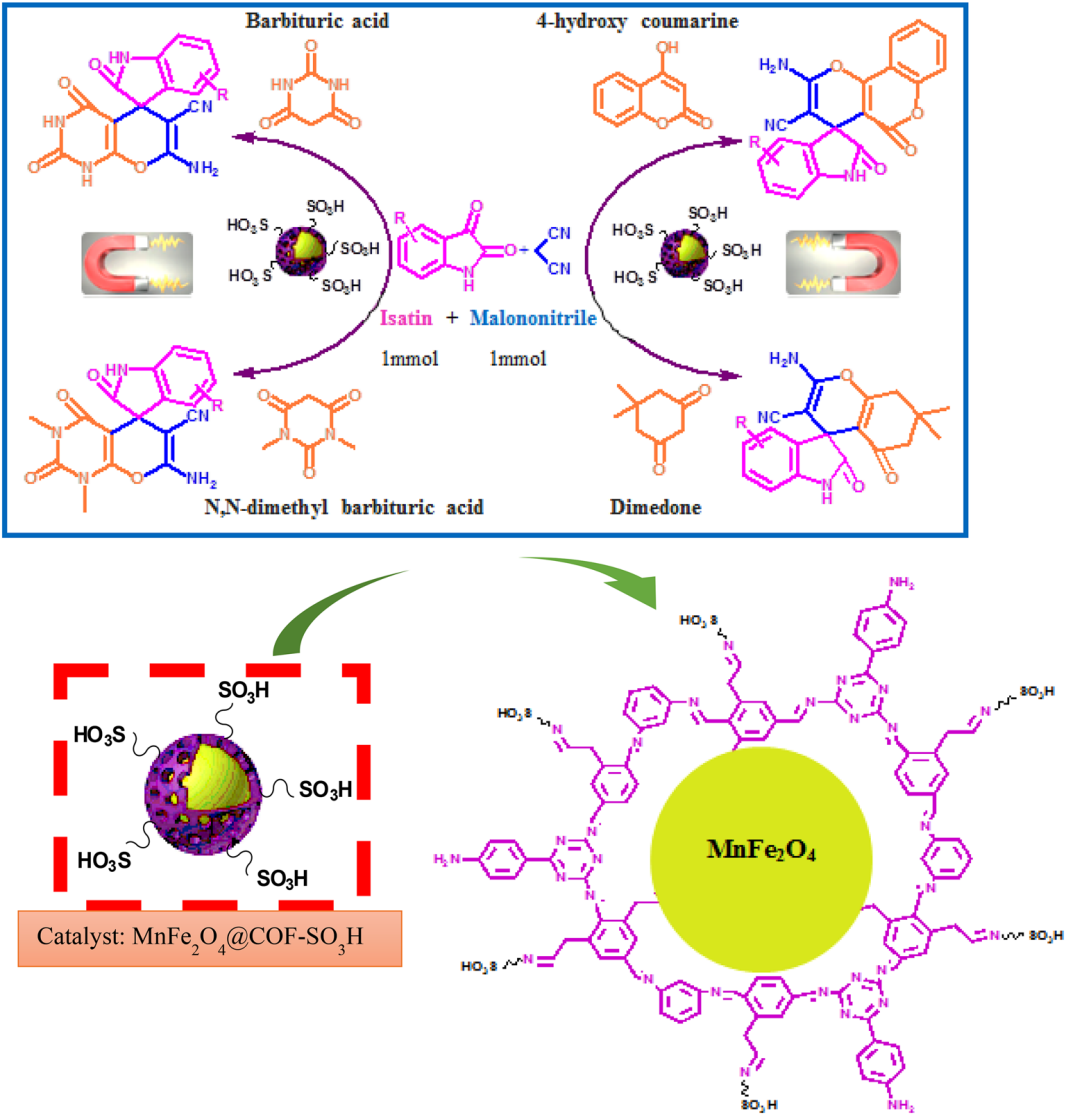
MnFe_2_O_4_@COF–SO_3_H nanocomposite (15 mol%) catalyzed the synthesis of spirooxindoles derivatives via a three component reaction in ethanol.

This study introduces a straightforward approach to synthesizing MnFe_2_O_4_@COF-SO_3_H, a durable catalyst with a combination of inorganic and organic components, as illustrated in detail in the lower section of Scheme 1. This eco-friendly catalyst can be easily isolated using an external magnet and offers cost-effective recovery, enabling its reuse for up to 5 cycles without compromising catalytic efficiency. It demonstrates remarkable efficacy in facilitating the synthesis of spiroxindole derivatives via a one-pot three-component condensation reaction utilizing malononitrile (1 mmol), isatin (1 mmol), and various 1,3-dicarbonyl compounds (1 mmol) under reflux conditions in EtOH (Scheme 1).

## Experimental section

### Structural analysis of the MnFe_2_O_4_@COF–SO_3_H nanocatalyst

Initially, the synthesis of manganese ferrites (MnFe_2_O_4_) nanoparticles involved the combination of Fe(III) salt and Mn(II) salt in an alkaline solution, resulting in the precipitation of spinel ferrite, MnFe_2_O_4_, from the solution.

Subsequently, magnetic covalent organic frameworks (MnFe_2_O_4_@COF) were constructed by employing MnFe_2_O_4_ nanoparticles along with melamine (MA) and terephthalaldehyde (TPA). Finally, chlorosulfonic acid was used to sulfonate the MnFe_2_O_4_@COF.

**Figure 2:**
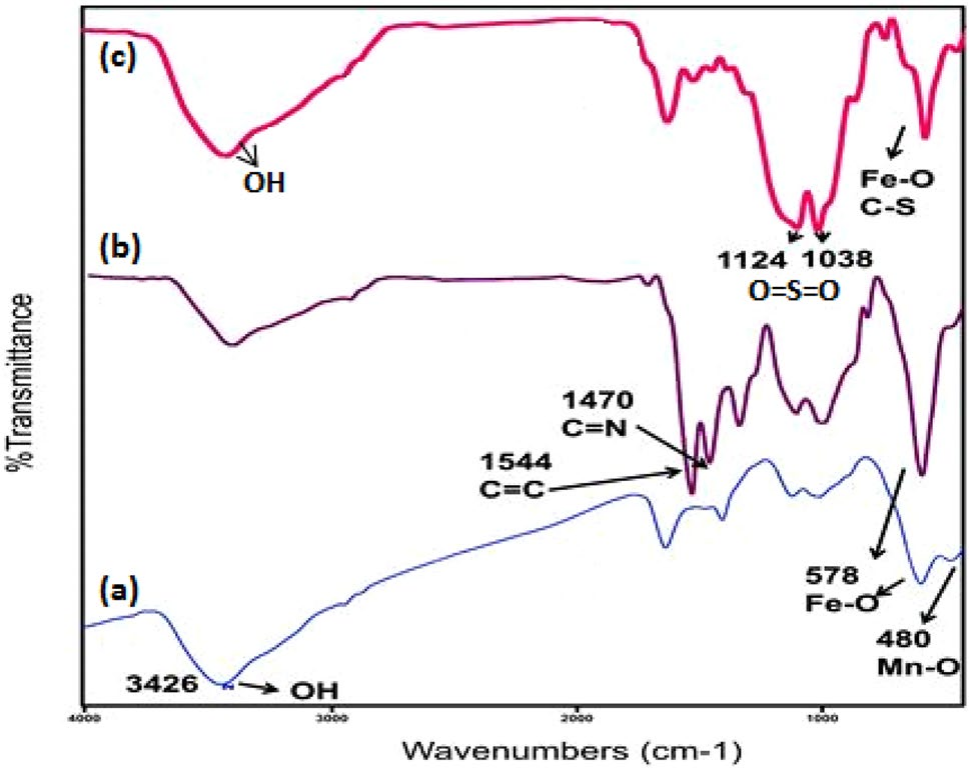
The FT-IR spectra of (**a**) MnFe_2_O_4_, (**b**) MnFe_2_O_4_@COF and (**c**) MnFe_2_O_4_@COF–SO_3_H.

Figure 2a–c illustrate the FT-IR spectra of MnFe_2_O_4_, MnFe_2_O_4_@COF, and MnFe_2_O_4_@COF-SO_3_H. The FT-IR spectrum depicted in Fig. 2a displays two distinct vibration bands at approximately 578 and 480 cm^−1^, which correspond to the Fe–O and Mn–O vibrational modes in manganese ferrite, respectively. This confirms the formation of a single-phase MnFe_2_O_4_ as no other metal oxide bands are observed within the 400–1000 cm^−1^ range. The broad peak observed at 3426 cm^−1^ is attributed to the stretching vibration of N–H functional groups or hydrogen-bonded surface water molecules.

The FT-IR spectra of MnFe_2_O_4_@COF revealed distinct differences when compared to those of MnFe_2_O_4_ nanoparticles and monomers (Fig. 2b). The characteristic aromatic rings can be detected at 1548 and 1474 cm^−1^, indicating the presence of C=C and C=N vibrations, while the absence of the C=O vibration of TPA at 1694 cm^−1^ confirmed that the COF shells were successfully formed through a Schiff-base reaction and coated onto the surface of MnFe_2_O_4_ nanoparticles.

The spectrum of MnFe_2_O_4_@COF-SO_3_H shows bands in the range of 1020–1150 cm^−1^, which correspond to the O=S=O asymmetric and symmetric stretching modes (Fig. 2c). The peak intensity in the range of 3400 has considerably increased, providing strong evidence for the presence of the acidic group. These findings indicate that –SO_3_H groups have been effectively incorporated into the primary polymeric framework of COF. Furthermore, it is worth noting that the successful functionalization of MnFe_2_O_4_@COF with –SO_3_H groups can be confirmed through EDX analysis.

**Figure 3:**
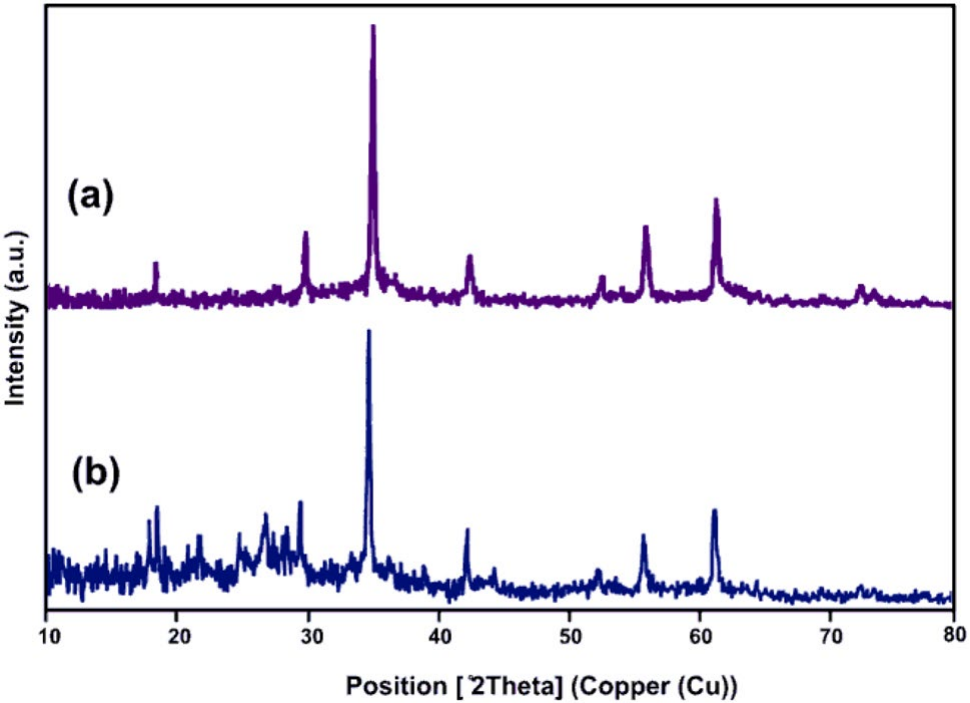
The X-ray diffraction (XRD) patterns of (**a**) MnFe_2_O_4_, and (**b**) MnFe_2_O_4_@COF–SO_3_H.

Figure 3 presents the XRD spectra of MnFe_2_O_4_ and MnFe_2_O_4_@COF–SO_3_H. Subfigure (a) of Fig. 3 displays the XRD pattern of MnFe_2_O_4_ nanoparticles, showing well-defined and intense peaks indicating good crystallinity. The diffraction peaks observed at 18.68°, 29.69°, 34.97°, 42.53°, 56.18° and 61.61° correspond to the lattice planes of (111), (220), (311), (400), (511) and (440), respectively, in agreement with standard JCPDS (No. 01-074-2403). These peaks correspond to specific lattice planes, confirming the phase purity of MnFe_2_O_4_. No impurity peaks were detected. The grain size for the high-intensity peaks was approximately 20 nm, determined using Scherrer’s equation. In the XRD pattern (Fig. 3b), broad peaks between 2*θ* values of 15°–30° suggest the amorphous nature of the porous organic polymer. However, the surface structure of the material remained relatively unchanged before and after modification with sulfonic acid groups, indicating that the original structure was not significantly altered.

**Figure 4:**
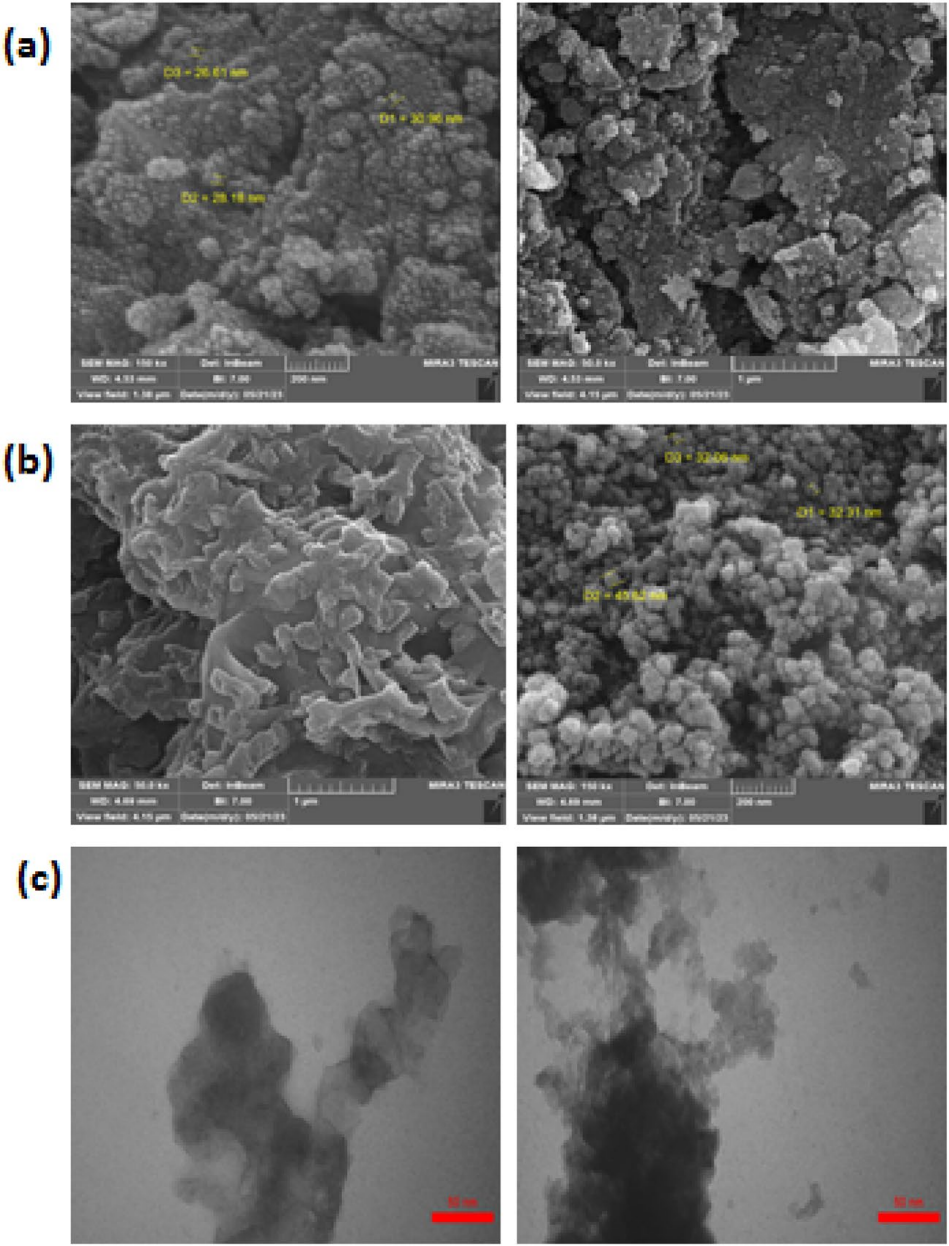
The FESEM images of (**a**) MnFe_2_O_4_, (**b**) MnFe_2_O_4_@COF-SO_3_H, (**c**) TEM images of MnFe_2_O_4_@COF–SO_3_H.

The MnFe_2_O_4_ and MnFe_2_O_4_@COF-SO_3_H materials were examined using FE-SEM analysis to investigate their morphological and structural properties (Fig. 4a, b). The obtained images indicate that the particles exhibit a nearly spherical morphology. It is evident from the images that both MnFe_2_O_4_ and MnFe_2_O_4_@COF-SO_3_H possess nano-sized structures, with average sizes of approximately 28 nm and 35 nm, respectively. Additionally, partial aggregation can be observed, which is beneficial for applications requiring high electron transfer conductivity.

To further examine the catalyst's morphology, a TEM analysis was conducted. The TEM images of the catalyst under investigation indicate a nearly uniform distribution of MnFe_2_O_4_ nanoparticles within the acidic COF, as shown in Fig. 4c.

**Figure 5:**
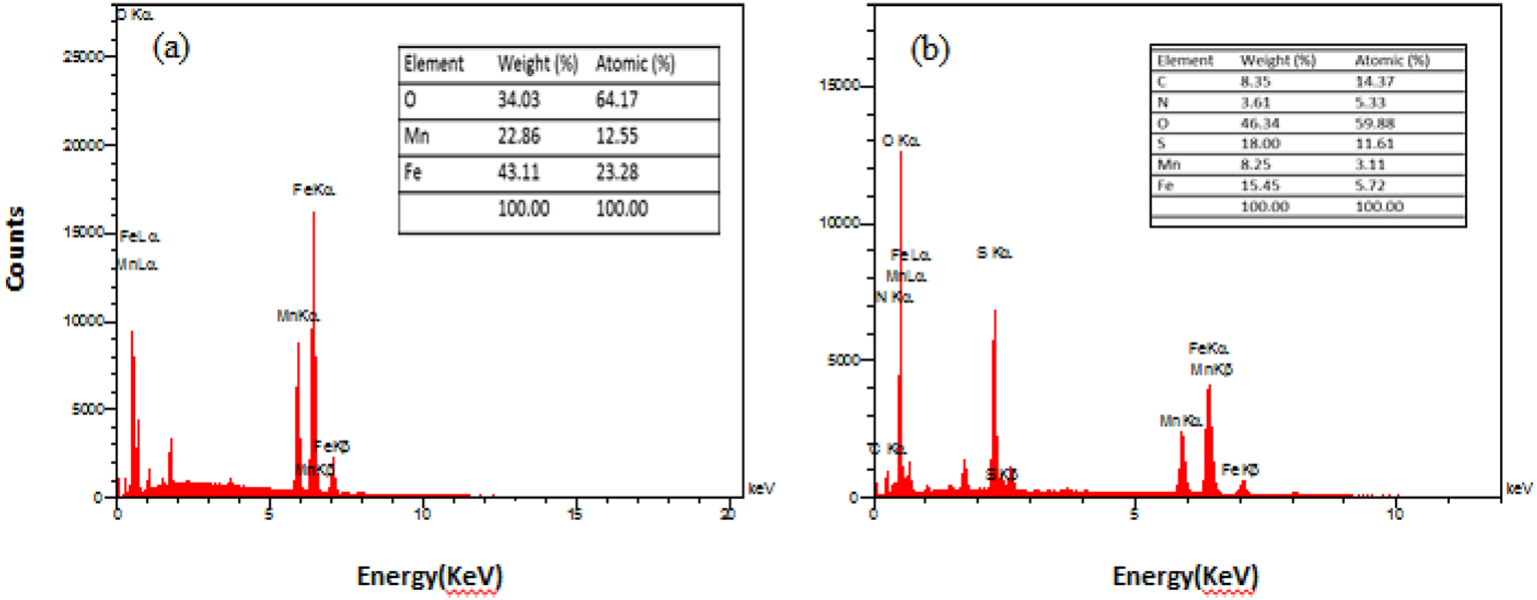
The EDX spectra of (**a**) MnFe_2_O_4_, (**b**) MnFe_2_O_4_@COF–SO_3_H.

**Figure 6:**
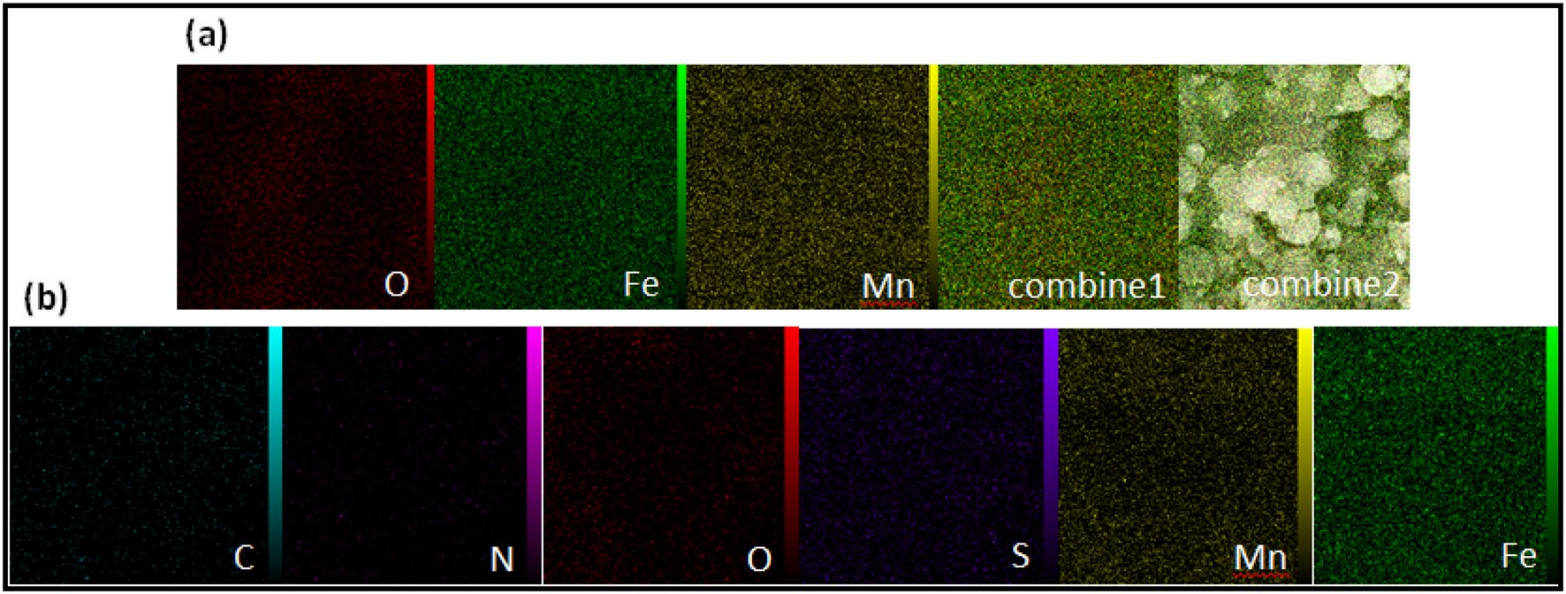
Elemental mapping of (**a**) MnFe_2_O_4_, and (**b**) MnFe_2_O_4_@COF–SO_3_H.

The EDX results confirm the successful preparation of pure MnFe_2_O_4_ nanoparticles without any significant impurities detected in the sample (Fig. 5a). Furthermore, the presence of Mn, N, Fe, O, C, and S species in the MnFe_2_O_4_@COF-SO_3_H material is confirmed by EDX analysis (Fig. 5b). The sulfur loading content is determined to be 18 wt%, providing further evidence that sulfonic acid was successfully loaded onto the polymer surface. EDS mapping images demonstrate that all elements are uniformly dispersed within the polymer network in both samples (Fig. 6a, b).

**Figure 7:**
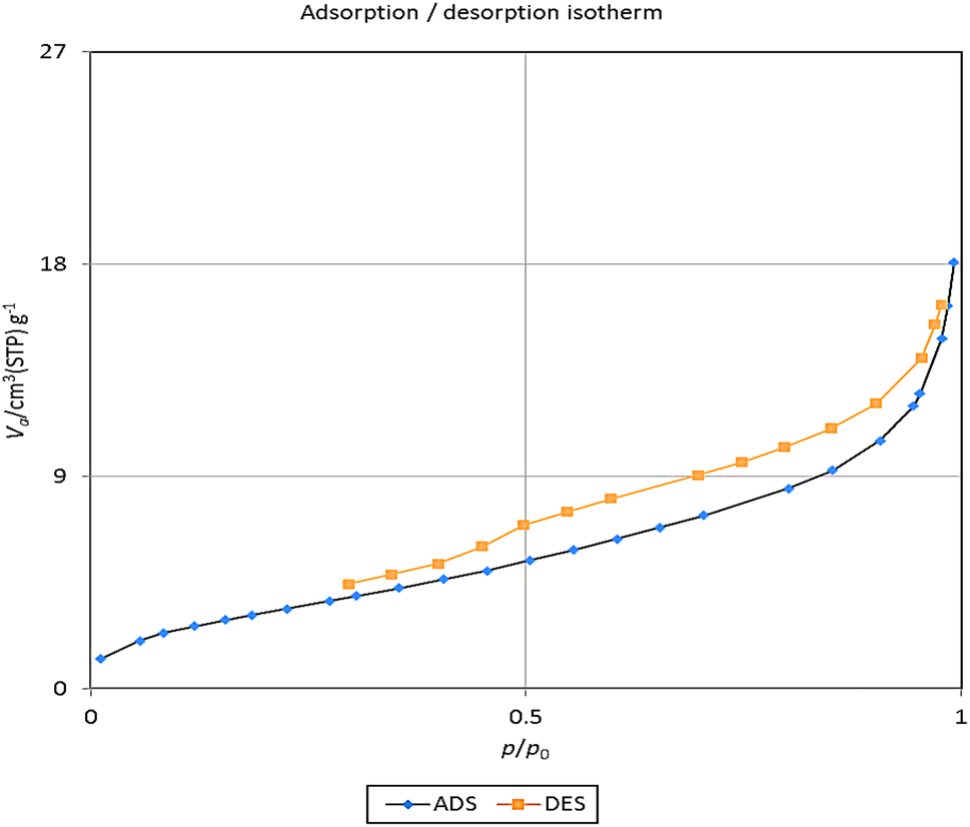
The BET image of MnFe_2_O_4_@COF–SO_3_H.

Porous structures have a critical role in regulating catalytic properties within an academic context. Additionally, these structures often display intricate and interconnected three-dimensional geometries. to determine the porous characteristics of the sample, N_2_ adsorption–desorption analysis was conducted at 77 K (Fig. 7). The N_2_ isotherm of the MnFe_2_O_4_@COF–SO_3_H catalyst confirmed a mesoporous structure, indicated by its type IV isotherm. Also, H2 type hysteresis loop in the relative pressure ranges from 0.3 to 1.00, is attributed to mesopore materials.

The specific surface area of the catalyst was determined to be approximately 12.671 m^2^ g^−1^. Furthermore, the composite exhibited a pore size distribution of approximately 8.60 nm and a total pore volume of 0.027 cm^3^ g^−1^. Therefore, the fabricated MnFe_2_O_4_@COF–SO_3_H catalyst demonstrated a suitable pore structure and a favorable surface area, which could significantly enhance its catalytic efficiency.

**Figure 8:**
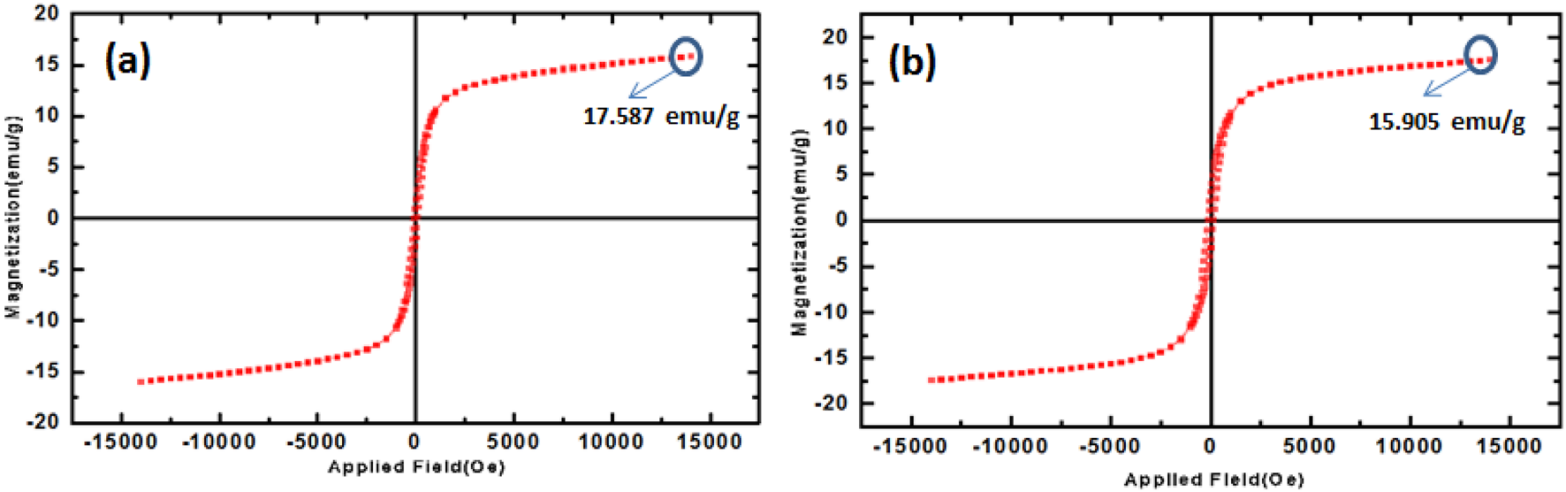
The magnetic hysteresis curves of (**a**) MnFe_2_O_4_ and (**b**) MnFe_2_O_4_@COF at room temperature.

The magnetic hysteresis curves of MnFe_2_O_4_ and MnFe_2_O_4_@COF at room temperature are illustrated in Fig. 8. The saturation magnetization of these materials was measured as 17.58 and 15.90 emu g^−1^, respectively. The lower saturation magnetization observed can be attributed to various factors, including crystalline nature, particle size, particle arrangement, adsorbed layer of molecules on the particle surface, and random canting of particle surface spins^36^. This dependence is also influenced by the concentration of trivalent and divalent cations in the tetrahedral and octahedral sites^37^. Furthermore, nanosized particles with a high surface-to-volume ratio exhibit decreased saturation magnetization.

**Figure 9:**
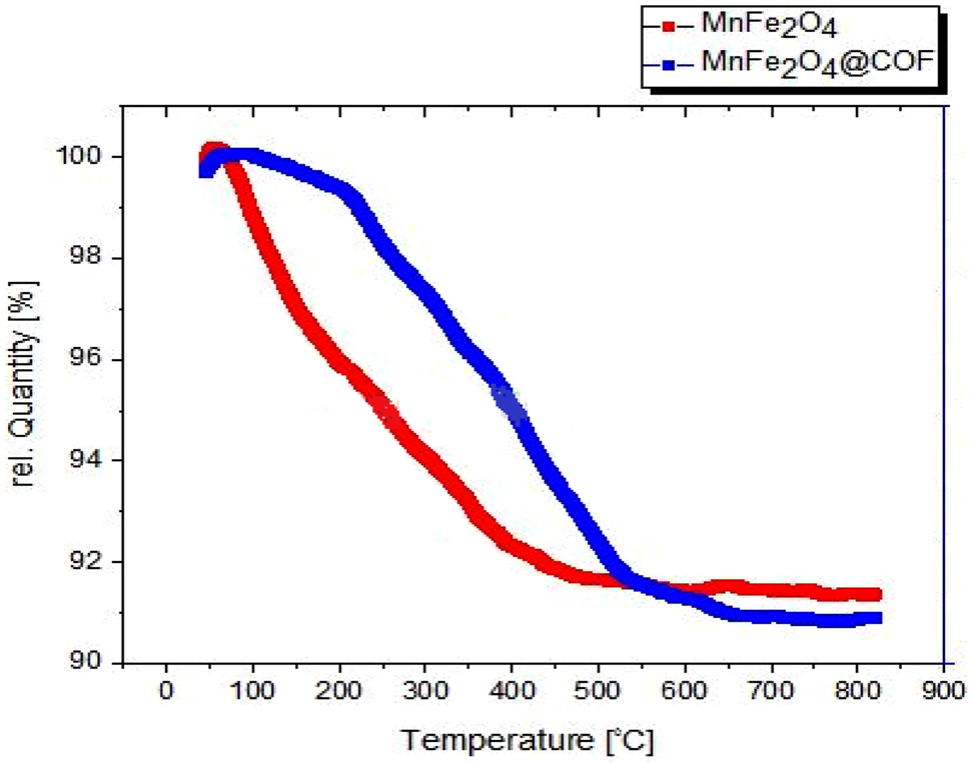
The TGA curve of MnFe_2_O_4_ and MnFe_2_O_4_@COF–SO_3_H.

The thermal behavior of MnFe_2_O_4_@COF–SO_3_H nanoparticles was analyzed with TGA. The MnFe_2_O_4_ nanoparticles showed a weight loss of 8.1% up to 500 °C, which was attributed to the evaporation and breakdown of small organic compounds, as seen in Fig. 9. In contrast, MnFe_2_O_4_@COF displayed two thermal degradation stages on its hydrogenation curve. The initial weight loss of 1% occurred within the temperature range of 100–250 °C, resulting from the evaporation of bound water and volatile small organic compounds. Subsequently, a gradual weight loss took place between 250 and 500 °C as a consequence of the sulfonic acid group being broken down first, followed by the decomposition of COF's organic structure.

**Figure 10:**
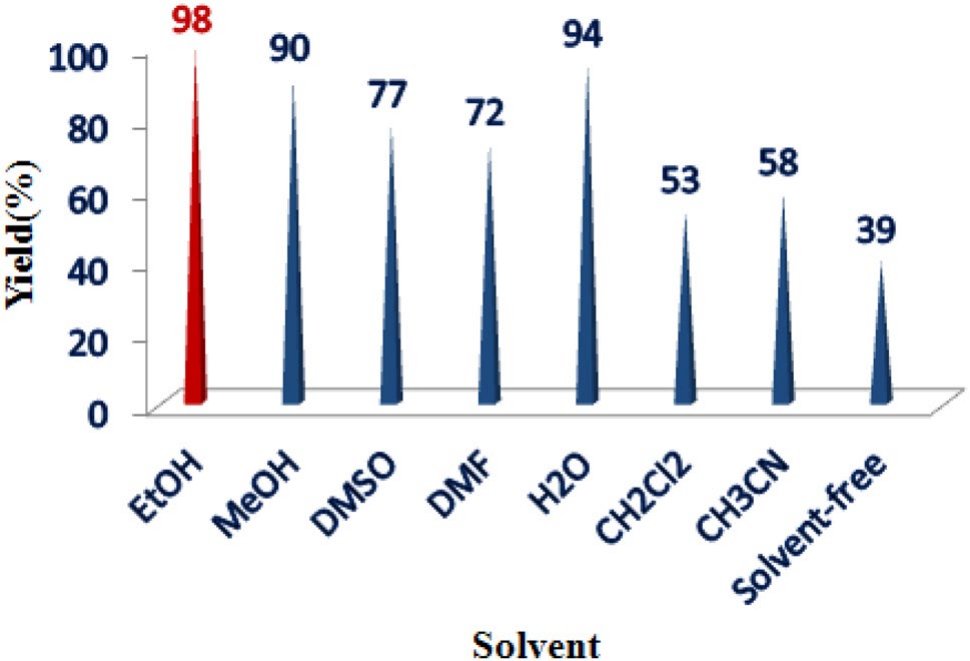
The impact of various solvents on the preparation of oxindole using MnFe_2_O_4_@COF–SO_3_H.

The efficacy of synthesized nanocatalysts in organic reactions was demonstrated through the utilization of COF-SO_3_H immobilized on MnFe_2_O_4_ nanoparticles [MnFe_2_O_4_@COF-SO_3_H] as an effective and reusable nanocatalyst for the production of spirooxindoles. This was achieved by coupling 1,3‐dicarbonyls with malononitrile and various isatins. To evaluate the reaction, dimedone, malononitrile, and isatins were utilized as model substrates in a variety of solvents (Fig. 10). The results revealed that the efficacy of the reaction was impacted by different solvents. Low yields (53–58%) were obtained when acetonitrile and dichloromethane were used as solvents, whereas water, DMF, and DMSO improved yields. Ethanol was found to be the most effective solvent, producing a yield of 98%, exceeding all other solvents tested. Without solvent, the yield decreased to 39% for model reactions.

The use of isatin in electrophilic reactions with diverse nucleophiles is considered a fundamental approach for generating multicomponent reactions. In addition to its role as a solvent, protic solvents can facilitate the enolization of dimedone by forming hydrogen bonds with the OH group. This, in turn, enhances the nucleophilic properties of the methylene carbon (C-2) of dimedone and results in an accelerated reaction rate^38,39^.

**Figure 11:**
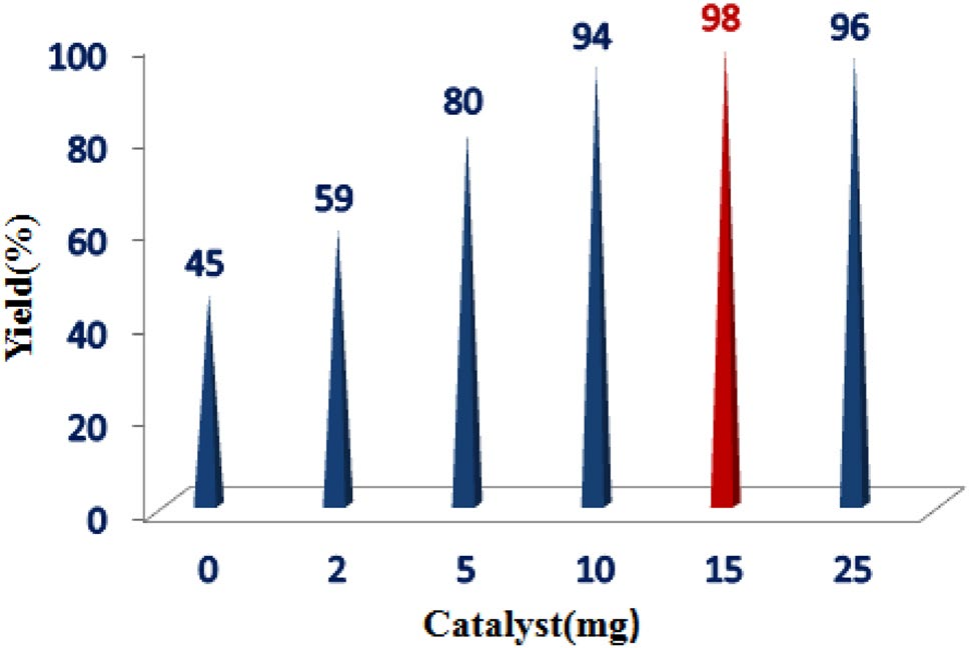
The effect of varying quantities of MnFe_2_O_4_@COF–SO_3_H on the synthesis of oxindole.

Several experiments were carried out to regulate the quantity of catalyst employed. These experiments revealed that augmenting the catalyst quantity from 5 to 15 mol% resulted in a yield increase from 80 to 98%. However, employing a larger quantity of nanocatalysts (25 mol%) did not enhance the reaction yield, as evidenced in Fig. 11.

Different reaction conditions were investigated in the model reaction involving dimedone, malononitrile, and isatin, to obtain the highest production yield and demonstrate the catalyst's efficacy. According to the findings, carrying out the reaction at 80 °C with 15 mg of catalyst (MnFe_2_O_4_@COF–SO_3_H) in ethanol as the solvent increases production yield (Table 1, entry 5).

**Table 1:** The optimization of reaction conditions aimed at generating spiro-pyrano chromen.

Entry	Catalyst	Solvent/T (°C)	Catalyst (mg)	Time (min)	Yield^a^ (%)
1	MnFe_2_O_4_	Ethanol/80 °C	15	100	60
2	MnFe_2_O_4_@COF–SO_3_H	Ethanol/50 °C	15	30	71
3	MnFe_2_O_4_@COF–SO_3_H	Ethanol/70 °C	15	15	80
4	MnFe_2_O_4_@COF–SO_3_H	Solvent-free/80 °C	15	25	73
5	MnFe_2_O_4_@COF–SO_3_H	Ethanol/80 °C	15	8	98
6	MnFe_2_O_4_@COF–SO_3_H	Ethanol/90 °C	15	8	96
7	MnFe_2_O_4_@COF–SO_3_H	H_2_O/80 °C	15	40	65

^a^Isolated yields.

Table 2 presents the outcomes of the synthesis of different spiro[chromene-4,3'-indolines], spiro[indoline-3,4'-pyrano[3,2-c]chromene] and spiro[indoline-3,5'-pyrano[2,3-d]pyrimidine] compounds. The reactions were carried out in ethanol as a solvent at a temperature of 80 C using various 1,3-dicarbonyls and isatin derivatives.

**Table 2:** The synthesis of spirooxindole derivatives 3 using MnFe_2_O_4_@COF–SO_3_H.


Entry	R	1,3-Dicarbonyl [2(a-d)]	Product^b^	Time (min)	Yield^c^ (%)	M.P./M.P. (^o^C)^c^
**1**	H	3a		10	98	305–307/305–307^40^
**2**	Br	3a		12	98	304–306/305–307^40^
**3**	NO_2_	3a		10	96	303–305/302–304^40^
**4**	F	3a		15	94	> 300/> 300^41^
**5**	H	3b		10	93	> 300/> 300^42^
**6**	Br	3b		11	95	> 300/> 300^42^
**7**	NO_2_	3b		12	97	> 300/> 300^42^
**8**	F	3b		15	95	> 300/> 300^42^
**9**	H	3c		10	93	274–276/275–276^43^
**10**	NO_2_	3c		10	96	285–296/286–288^44^
**11**	F	3c		15	94	210–212/210^45^
**12**	H	3d		18	91	228–230/229–231^46^
**13**	Br	3d		12	97	260–262/259–260^43^
**14**	NO_2_	3d		12	95	282–285/282–284^47^
**15**	F	3d		15	92	225–227

### Proposed mechanism

**Scheme 2.**
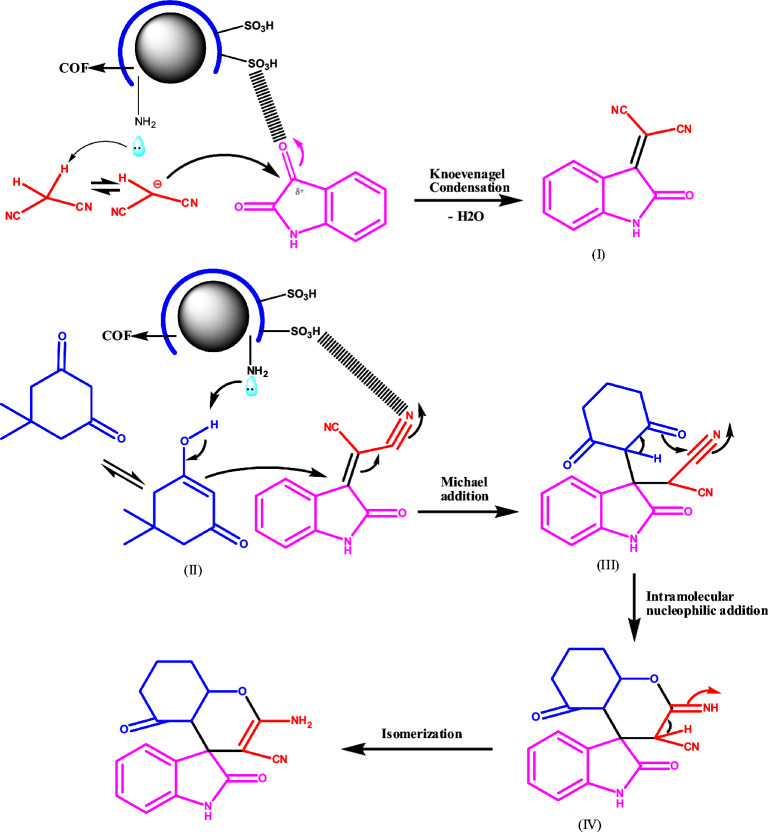
The illustrative mechanism utilized for the synthesis of spirooxindole using MnFe_2_O_4_@COF–SO_3_H nanocatalyst.

In Scheme 2, we have a way to make spiro compounds and it involves a process called the catalytic cycle. To create spirooxindole, we use isatin, cyclic 1,3-diketone, and malononitrile in a three-component reaction. The process happens in two steps: First, the SO_3_H nanocatalyst binds to the oxygen atom of the carbonyl group through electrostatic attraction. At the same time, lone pairs of the amino group of COF remove the acidic hydrogen from malononitrile. Then, carbanion attacks the carbonyl group of isatin and produces isatylidenemalononitrile (I) through a Knoevenagel condensation. In the following step, the carbanion reacts with the activated double bond of cyclic 1,3-dicarbonyl (II) via Michael addition and produces intermediate (III). Afterward, an intramolecular nucleophilic addition reaction forms an intermediate (IV). Lastly, we get our final product by an isomerization process.

### Reusability of the catalyst

**Figure 12:**
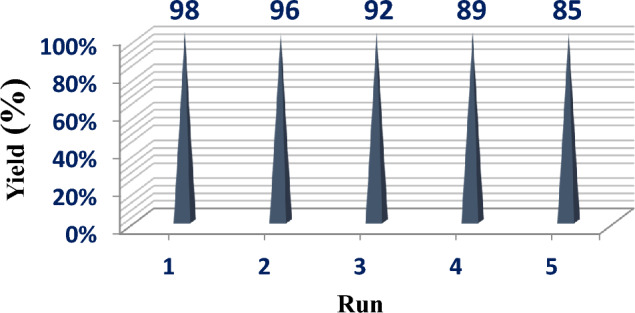
Recycling values for MnFe_2_O_4_@COF–SO_3_H.

To assess the possibility of reusing the catalyst, it was collected after the reaction process using an external permanent magnet. Subsequently, the catalyst was subjected to multiple washes with ethanol and dried at a temperature of 50 °C before its use in subsequent cycles. The findings indicate that the catalyst was effectively utilized in five consecutive reaction cycles with no notable decline in product yield, with initial yields of 98% and 85% by the fifth cycle (Fig. 12).

This catalyst was prepared using simple salts and cheap materials. Also, according to the economic approach, it can be used up to 5 times in the reaction with the greatest effect.

Results from this study and other studies on the model reaction show that our method, which uses a MnFe_2_O_4_@COF–SO_3_H catalyst, produces a higher yield in less time (Table 3). As depicted in Table 3, the use of other catalysts requires a longer time, lower efficiency, and higher costs^48–50^. In contrast, utilizing MnFe_2_O_4_@COF–SO_3_H solves these problems considering that it is possible to collect the catalyst with an external magnet. The recovery of the catalyst is easily possible without loss of efficiency, resulting in significant cost reductions. Moreover, this approach is also considered environmentally favorable.

**Table 3:** Comparison the various catalysts for the synthesis of spirooxindole compounds.

Entry	Catalyst	Solvent	Time	Yield (%)	References
1	Fe_3_O_4_@APTPOSS MNPs	–	45 h	88	^43^
2	Sulfated choline-based heteropolyanion	EtOH	45 h	91	^51^
3	Cesium fluoride	EtOH	5 h	82	^52^
4	Nickel oxide nanoparticles	H_2_O	5 h	98	^53^
5	Amino-appended b-cyclodextrin	H_2_O	7 h	91	^54^
6	(DABCO)@mesoporous silica SBA-15	H_2_O	10 h	96	^55^
7	[Bmim]BF_4_	SF	3 h	94	^56^
8	THAM	EtOH	4 h	94	^57^
9	TiO_2_-CNTs	H_2_O	6 h	94	^58^
10	Sg-C_3_N_4_ (20 wt%)	H_2_O	10 min	96	^59^
11	MnFe_2_O_4_@COF–SO_3_H	EtOH	15 min	98	This work

### Substances and methods

All reagents and solvents used in the study were commercially purchased and did not undergo any additional purification. Fourier transform infrared (FT-IR) spectroscopy was conducted using a Nicolet Magna-400 spectrometer with KBr pellets. 1H NMR data were collected in DMSO-d6 using a Bruker DRX-400 spectrometer and tetramethylsilane as the internal reference. XRD patterns were recorded using a Philips diffractometer with monochromatized Cu K radiation. The morphology of the nanoparticles was analyzed using field emission scanning electron microscopy (FE-SEM) with model MIRA3. An Arya Electron Optic instrument was utilized in the academic analysis of the catalyst through the use of electron dispersive X-rays (EDX) and mapping techniques. The surface area measurement was conducted using the Brunauer Emmett Teller (BET) method, which involved nitrogen adsorption analyzed by a mechanized gas adsorption analyzer (Belsorp mini II, Microtrac Bel Corp). The microscopic morphology of the nanoparticles was observed using a Philips transmission electron microscope (TEM) operating at 100 Kv. At Iran's Kashan University, the magnetic characteristics of materials were evaluated with a magnetometer (VSM, PPMS-9T) at a temperature of 300 K. Thermogravimetric analysis (TGA) was performed on a Mettler TA4000 system TG-50, utilizing a heating rate of 10 K min1 in an N2 atmosphere. The Yanagimoto micro melting point device was employed to measure the melting points without any correction. To monitor the reaction and determine substrate purity, thin-layer chromatography (TLC) was carried out on silica-gel polygram SILG/UV 254 plates provided by the Merck Company.

### Synthesis of catalyst

#### Preparation of modified MnFe_2_O_4_ nanoparticles

After 30 min of nitrogen gas bubbling in 200 mL of purified, deoxygenated water, 5 g of Mn(NO_3_)_2_·4H_2_O and 14 g of Fe(NO_3_)_3_·6H_2_O were dissolved in ultrapure water with vigorous mechanical stirring. The aforementioned mixture was then stirred while 2.0 M NaOH solution was added dropwise until the pH reached 11. After that, the mixture was heated to 100 °C and maintained there for 2 h. In an external magnetic field, a black precipitate was gathered and then cleaned with ultrapure water. To get rid of the contaminants connected with the operations (such as OH^−^, NO^3−^, and Na^+^), this washing was done three times. After freeze-drying, pure MnFe_2_O_4_ nanoparticles were finally produced.

#### Preparation of MnFe_2_O_4_@COF

The synthesis of magnetic covalent organic frameworks (MnFe_2_O_4_@COF) involved dissolving 0.20 g of MnFe_2_O_4_ nanoparticles in a solution of 50 mL DMSO, 2 mmol of melamine (MA), and 3 mmol of terephthalaldehyde (TPA). Following a 30-min sonication of the combination, the homogeneous black suspension was placed in a stainless-steel autoclave lined with Teflon, which was then heated to 180 °C for a reaction time of 24 h. The produced MnFe_2_O_4_@COF was magnet-separated, followed by three rounds of washing in tetrahydrofuran, anhydrous methanol, and dichloromethane. The next step was to vacuum-dry the produced MnFe_2_O_4_@COF at 50 °C.

### Synthesis of MnFe_2_O_4_@COF‑SO_3_H

Chlorosulfonic acid was utilized to sulfonate the MnFe_2_O_4_@COF. A standard synthesis involved suspending 0.5 g of the MnFe_2_O_4_@COF in 20 ml chloroform in a 25-mL round-bottomed flask, followed by the dropwise addition of 2 ml of chlorosulfonic in CH_2_Cl_2_ (10 mL) throughout 2 h at room temperature. The catalyst obtained was subjected to multiple washes with chloroform and subsequently dried for 24 h at 60 °C in an oven.

### Modus operandi for the synthesis of spiro[chromene-4,3'-indolines], spiro[indoline-3,4'-pyrano[3,2-c]chromene] and spiro[indoline-3,5'-pyrano[2,3-d]pyrimidine] compounds

Isatin derivatives (1 mmol), 4-hydroxycoumarin or dimedone or barbituric acid (1 mmol), and malononitrile (1 mmol) with 15 mg of the catalyst (MnFe_2_O_4_@COF-SO_3_H) were added to a 50 ml round-bottom flask and mixed at 80 °C in the presence of ethanol solvent until the reaction was fully developed and its progress assessed through thin-layer chromatography. To create the pure compounds, the obtained products were dried and then washed with ethanol.

### Analysis and characterization of the synthesized compounds

Compound 4*f* exhibits an absorption signal at 3439 cm^−1^ in its IR spectrum, indicating the presence of an NH group in the molecule's structure. The absorption peaks observed at 1782, 1726, and 1665 cm^−1^ can be assigned to the stretching vibrations of carbonyl groups. Additionally, a C=C stretching band is observed at 1622 cm^−1^.

When analyzing the compound's 1HNMR spectrum, a singlet signal is detected at δ = 10.93 ppm for the NH proton. The hydrogens of aromatic moieties produce signals within the range of δ = 7.53–6.89 ppm. In dimedone, the hydrogens of 2CH_2_ appear as two doublet peaks at δ = 2.20 and δ = 2.06 ppm with 16 Hz, along with a single peak at δ = 2.95 ppm. The two sharp singlet peaks observed at δ = 1.13 and δ = 1.09 ppm can be attributed to the presence of the 2CH_3_ groups in the dimedone moiety.

## Conclusion

This report discussed the preparation of COF and their composites with magnetic nanoparticles (MnFe_2_O_4_). These materials possess distinct characteristics that make them as viable options for material science applications. Firstly, the synthesis process follows a one-pot approach. Secondly, the materials offer customizable porosity. Thirdly, the starting components of the materials are inexpensive. Fourthly, the catalyst can be isolated with an external magnet. Lastly, nanocomposites with elevated amounts of nitrogen have been successfully produced. MnFe_2_O_4_@COF-SO_3_H serves as a reusable and efficient nanocatalyst for the synthesis of spirooxindoles, comparable to other commonly used catalysts. The proposed method suggests various advantages, such as simplicity, high yields, shorter reaction time, reduced environmental impact, and a safe and cost-effective starting procedure (no toxic solvents were used in the reaction or work-up procedures). Consequently, this method is valuable and appealing for the preparation of these important compounds. Additionally, considering the abundance of isatins and 1,3-dicarbonyl compounds, this approach holds the potential for generating libraries with significant diversity. Therefore, it is anticipated that this method will find widespread application in drug discovery and combinatorial chemistry.

## Supplementary Information

Supplementary Information.

## Data Availability

In terms of data availability, all the data generated or analyzed during this study can be found in the published article and its supplementary information file.
