# Supplementary material for: Synthesis of COF-SO3H immobilized on manganese ferrite nanoparticles as an efficient nanocomposite in the preparation of spirooxindoles
Source: Sci Rep. 2023 Dec 20;13:22731. doi: 10.1038/s41598-023-49628-7 (PMC10733289; doi:10.1038/s41598-023-49628-7)
Supplement: Supplementary file 1 — Supplementary Information. [file 41598_2023_49628_MOESM1_ESM.docx]

**Synthesis of COF-SO_3_H immobilized on Manganese Ferrite Nanoparticles as an efficient nanocomposite in the preparation of spirooxindoles**

Samira Moein Najafabadi^1^, Javad Safaei-Ghomi*^1^

^1^Department of Organic Chemistry, Faculty of Chemistry, University of Kashan, Kashan, I. R. Iran,

*E-mail address: [safaei@kashanu.ac.ir](mailto:safaei@kashanu.ac.ir).

**Characterization data**

**Physical and spectroscopic data of all products**

**2-amino-7,7-dimethyl-2',5-dioxo-5,6,7,8-tetrahydrospiro[chromene-4,3'-indoline]-3-carbonitrile (4a):** white powder**;** IR (KBr,cm^-1^): 3374, 3311 (NH_2_), 3144 (NH), 2962 (CH stretch), 2192 (CN), 1722,1657 ( C=O stretch), 1608, 1469(C=C aromatic stretch), 1345 (C-N), 1219, 1053 (2 C-O); ^1^H NMR (DMSO-d_6_, 400 MHz) δ (ppm): 1.01 (s, 3H, CH3), 1.04 (s, 3H, CH3**),** 2.08-2.20 (m, 2H, CH2), 2.56 (d, J=4.4 Hz, 2H, CH_2_), 6.79 (d, *J*= 7.6 Hz, 1H, ArH), 6.89-6.91 (t, 1H, ArH), 6.98 (d, J=7.2 Hz, 1H, ArH), 7.13-7.16 (m, 1H, ArH), 7.23 (brs, 2H, NH2), 10.40 (s, 1H, NH).

**2-amino-5'-bromo-7,7-dimethyl-2',5-dioxo-5,6,7,8-tetrahydrospiro[chromene-4,3'-indoline]-3-carbonitrile (4b):** white powder; IR (KBr,cm^-1^): 3395, 3289 (NH_2_), 3157 (NH), 2957 (CH stretch), 2193 (CN), 1726,1654 ( C=O stretch), 1606, 1471 (C=C aromatic stretch), 1347 (C-N), 1219, 1054 (2 C-O); ^1^H NMR (DMSO, 400 MHz) δ (ppm): ^1^H NMR (DMSO-d_6_, 400 MHz) δ (ppm): 1.03 (s, 6H, 2 CH3**),** 2.08-2.21 (m, 2H, CH2), 2.60 (d, J=18 Hz, 2H, CH_2_), 6.76 (d, *J*= 8 Hz, 1H, ArH), 7.21 (s, 1H, ArH), 7.31-7.33 (m, 3H, NH_2_, ArH), 10.55 (s, 1H, NH).

**2-amino-7,7-dimethyl-5'-nitro-2',5-dioxo-5,6,7,8-tetrahydrospiro[chromene-4,3'-indoline]-3-carbonitrile (4c):** white powder; IR (KBr,cm^-1^): 3390, 3253 (NH_2_), 3175 (NH), 2957 (CH stretch), 2189 (CN), 1740,1643 ( C=O stretch), 1590, 1467 (C=C aromatic stretch), 1520, 1342 (N=O), 1221, 1059 (2 C-O);^1^H NMR (DMSO, 400 MHz) δ (ppm); ^1^H NMR (DMSO-d_6_, 400 MHz) δ (ppm): 1.03 (s, 6H, 2 CH3), 2.09-2.22 (m, 2H, CH2), 2.55-2.69 (m, 2H, CH_2_), 7.03 (d, *J*=8.6 Hz, 1H, ArH), 7.46 (brs, 2H, NH2), 7.97 (d, J=2.8 Hz, 1H, ArH), 8.15 (dd, J=2.6, 8.6 Hz, 1H, ArH), 11.19 (s, 1H, NH).

**2-amino-5'-fluoro-7,7-dimethyl-2',5-dioxo-5,6,7,8-tetrahydrospiro[chromene-4,3'-indoline]-3-carbonitrile (4d):** IR (KBr,cm^-1^): white powder; 3374, 3298 (NH_2_), 3158 (NH), 2959 (CH stretch),2878 (-CHF), 2191 (CN), 1725,1654 ( C=O stretch), 1603, 1483 (C=C aromatic stretch),1348 (C-N), 1225, 1056 (2 C-O);^1^H NMR (DMSO, 400 MHz) δ (ppm): ^1^H NMR (DMSO-d_6_, 400 MHz) δ (ppm): 1.02 (s, 3H, CH3), 1.03 (s, 3H, CH3**),** 2.15 (m, 2H, CH2), 2.56 (s, 2H, CH_2_), 6.76-6.82 (m, 1H, ArH), 6.96-7.00 (m, 2H, ArH), 7.30 (brs, 2H, NH2), 10.42 (s, 1H, NH).

**2'-amino-2,5'-dioxo-5'H-spiro[indoline-3,4'-pyrano[3,2-c]chromene]-3'-carbonitrile (4e):** white powder; IR (KBr,cm^-1^): 3371, 3302 (NH_2_), 3201 (NH), 3050 (CH stretch), 2202 (CN), 1718,1670 ( C=O stretch), 1606, 1470 (C=C aromatic stretch), 1357 (C-N), 1218, 1082 (2 C-O);^1^H NMR (DMSO-d6, 400 MHz) δ (ppm): ^1^H NMR (DMSO-d_6_, 400 MHz) δ (ppm): 6.87 (d, J=7.6 Hz, 1H, ArH), 6.94 (t, J=7.6 Hz, 1H, ArH**),** 7.20-7.24 (m, 2H, ArH), 7.50 (t, J=8.4 Hz, 1H, ArH), 7.56 (d, J=8 Hz, 1H, ArH), 7.70 (brs, 2H, NH2), 7.75 (td, J=1.6, 8.4 Hz, 1H, ArH), 7.95 (dd, J=1.6, 7.6 Hz, 1H, ArH), 10.69 (s, 1H, NH).

**2'-amino-5-bromo-2,5'-dioxo-5'H-spiro[indoline-3,4'-pyrano[3,2-c]chromene]-3'-carbonitrile (4f):** light purple powder; IR (KBr,cm^-1^): 3331 (NH_2_), 3203 (NH), 2202 (CN), 1718,1671 ( C=O stretch), 1609, 1471 (C=C aromatic stretch), 1361 (C-N), 1222, 1088 (2 C-O);^1^H NMR (DMSO-d6, 400 MHz) δ (ppm): 6.84 (d, J=8.4 Hz, 1H, ArH), 7.40 (dd, J=2.4, 8.4 Hz, 1H, ArH**),** 7.50-7.57 (m, 3H, ArH), 7.75 (brs, 2H, NH2), 7.79 (d, J=8 Hz, 1H, ArH), 7.94 (d, J=8 Hz, 1H, ArH),ArH), 10.83 (s, 1H, NH).

**2'-amino-5-nitro-2,5'-dioxo-5'H-spiro[indoline-3,4'-pyrano[3,2-c]chromene]-3'-carbonitrile (4g):** white powder; IR (KBr,cm^-1^): 3380,3316 (NH_2_), 3208 (NH), 2201 (CN), 1740,1672 ( C=O stretch), 1610, 1469 (C=C aromatic stretch), 1521, 1354 (N=O), 1214, 1099 (2 C-O); ^1^H NMR (DMSO-d6, 400 MHz) δ (ppm: 7.09 (d, J=8.8 Hz, 1H, ArH), 7.51 (d, J=8.4 Hz, 1H, ArH**),** 7.57 (t, J=7.6 Hz, 1H, ArH), 7.79 (t, J=7.6 Hz, 1H, ArH), 7.85 (brs, 2H, NH2), 7.96 (d, J=8 Hz, 1H, ArH), 8.21 (dd, J=2.4, 8 Hz, 1H, ArH), 8.36 (d, J=2.4 Hz, 1H, ArH) 11.42 (s, 1H, NH).

**2'-amino-5-fluoro-2,5'-dioxo-5'H-spiro[indoline-3,4'-pyrano[3,2-c]chromene]-3'-carbonitrile (4h):** white powder; IR (KBr,cm^-1^): 3369,3296 (NH_2_), 3202 (NH), 2202 (CN), 1719,1670 ( C=O stretch), 1606, 1480 (C=C aromatic stretch), 1360 (C-N), 1227, 1108 (2 C-O); ^1^H NMR (DMSO-d6, 400 MHz) δ (ppm): 6.83-6.86 (m, 1H, ArH), 7.02-7.07 (td, J=2.8,6.8 Hz, 1H, ArH**),** 7.25-7.27 (dd, J=2.8,8.4 Hz, 1H, ArH), 7.51 (d, J=8.4 Hz, 1H, ArH), 7.57-7.58 (t, J=8 Hz, 1H, ArH), 7.73 (brs, 2H, NH2), 7.76-7.80 (td, J=1.6, 7.2 Hz, 1H, ArH), 7.95 (dd, J=1.6, 8 Hz, 1H, ArH), 10.70 (s, 1H, NH).

**7'-amino-2,2',4'-trioxo-1',2',3',4'-tetrahydrospiro[indoline-3,5'-pyrano[2,3-d]pyrimidine]-6'-carbonitrile (4i):** white powder; IR (KBr,cm^-1^): 3352,3303 (NH_2_), 3142 (NH), 2831 (-CH), 2203 (CN), 1722,1674 ( C=O stretch), 1465 (C=C aromatic stretch), 1334 (C-N), 1241, 1109 (2 C-O); ^1^H NMR (DMSO-d6, 400 MHz) δ (ppm): 6.78 (d, J=7.6 Hz, 1H, ArH), 6.91 (t, J=7.2 Hz, 1H, ArH**),** 7.13 (d, J=7.2 Hz, 1H, ArH), 7.17 (d, J=7.6 Hz, 1H, ArH), 7.37 (brs, 2H, NH2), 10.42, 11.12, 12.30 (s, 3H, 3NH).

**7'-amino-5-nitro-2,2',4'-trioxo-1',2',3',4'-tetrahydrospiro[indoline-3,5'-pyrano[2,3-d]pyrimidine]-6'-carbonitrile (4j):** white powder; IR (KBr,cm^-1^): 3439,3301 (NH_2_), 3205 (NH), 2939 (-CH), 2199 (CN), 1749,1691 ( C=O stretch), 1521, 1395 (N=O), 1465 (C=C aromatic stretch), 1332 (C-N), 1239, 1046 (2 C-O); ^1^H NMR (DMSO-d6, 400 MHz) δ (ppm): 7.01 (d, J=8.8 Hz, 1H, ArH), 7.57 (brs, H, NH2**),** 8.15-8.18 (dd, J=2.4, 8.4 Hz, 1H, ArH), 8.25 (d, J=2 Hz, 1H, ArH), 11.19, 11.23, 12.40 (s, 3H, 3NH).

**7'-amino-5-fluoro-2,2',4'-trioxo-1',2',3',4'-tetrahydrospiro[indoline-3,5'-pyrano[2,3-d]pyrimidine]-6'-carbonitrile (4k):** light Pink powder; IR (KBr,cm^-1^): 3490,3352 (NH_2_), 3169 (NH), 3066 (-CH), 2216 (CN), 1748,1679 ( C=O stretch), 1484 (C=C aromatic stretch), 1323 (C-N), 1190, 1113 (2 C-O); ^1^H NMR (DMSO-d6, 400 MHz) δ (ppm): 6.75-6.78 (m, 1H, ArH), 6.98 (td, J=2.8, 9.2 Hz, 1H, ArH)**,** 7.15-7.18 (dd, J=2.8, 9.2 Hz, 1H, ArH), 7.48 (brs, 2H, NH2), 10.50, 11.15, 12.32 (s, 3H, 3NH).

**7'-amino-1',3'-dimethyl-2,2',4'-trioxo-1',2',3',4'-tetrahydrospiro[indoline-3,5'-pyrano[2,3-d]pyrimidine]-6'-carbonitrile (4l):** Pink powder; IR (KBr,cm^-1^): 3375,3302 (NH_2_), 3175 (NH), 2200 (CN), 1692, 1639 ( C=O stretch), 1490 (C=C aromatic stretch), 1340 (C-N), 1191, 1048 (2 C-O); ^1^H NMR (DMSO-d6, 400 MHz) δ (ppm): 3.02 (s, 3H, CH3), 3.38 (s, 3H, CH3**),** 6.80 (d, J=8 Hz, 1H, ArH), 6.91 (t, J=7.6 Hz, 1H, ArH), 7.12 (d, J=7.2 Hz, 1H, ArH), 7.16 (t, J=7.6 Hz, 1H, ArH), 7.52 (brs, 2H, NH2), 10.50 (s, 1H, NH).

**7'-amino-5-bromo-1',3'-dimethyl-2,2',4'-trioxo-1',2',3',4'-tetrahydrospiro[indoline-3,5'-pyrano[2,3-d]pyrimidine]-6'-carbonitrile (4m):** purple powder; IR (KBr,cm^-1^): 3405, 3340 (NH_2_), 3176 (NH), 2944 (-CH), 2198 (CN), 1695, 1633 ( C=O stretch), 1495 (C=C aromatic stretch), 1300 (C-N), 1194, 1045 (2 C-O); ^1^H NMR (DMSO-d6, 400 MHz) δ (ppm): 3.04 (s, 3H, CH3), 3.38 (s, 3H, CH3**),** 6.77 (d, J=8 Hz, 1H, ArH), 7.34 (dd, J=2, 8 Hz, 1H, ArH), 7.40 (d, J=2 Hz, 1H, ArH), 7.65 (brs, 2H, NH2), 10.65 (s, 1H, NH).

**7'-amino-1',3'-dimethyl-5-nitro-2,2',4'-trioxo-1',2',3',4'-tetrahydrospiro[indoline-3,5'-pyrano[2,3-d]pyrimidine]-6'-carbonitrile (4n):** white powder; IR (KBr,cm^-1^): 3482, 3351 (NH_2_), 3174 (NH), 2196 (CN), 1692,1635 ( C=O stretch), 1509, 1389 (N=O), 1338 (C-N), 1193,1072 (2 C-O); ^1^H NMR (DMSO-d6, 400 MHz) δ (ppm): 2.50 (s, 3H, CH3), 3.02 (s, 3H, CH3**),** 7.03 (dd, J=2, 8.8 Hz, 1H, ArH), 7.78 (brs, 2H, NH2), 8.15-8.25 (m, 2H, ArH), 11.26 (s, 1H, NH).

**7'-amino-5-fluoro-1',3'-dimethyl-2,2',4'-trioxo-1',2',3',4'-tetrahydrospiro[indoline-3,5'-pyrano[2,3-d]pyrimidine]-6'-carbonitrile (4o):** white powder; IR (KBr,cm^-1^): 3489, 3321 (NH_2_), 3169 (NH), 2195 (CN), 1701, 1641 ( C=O stretch), 1489 (C=C aromatic stretch), 1274, 1187 (2 C-O); ^1^H NMR (DMSO-d6, 400 MHz) δ (ppm): 3.03 (s, 3H, CH3), 3.38 (s, 3H, CH3**),** 6.77-6.80 (m, 1H, ArH), 6.97-7.02 (td, J=2.8, 8.4 Hz, 1H, ArH), 7.11 (dd, J=2.8, 8.4 Hz, 1H, ArH), 7.64 (brs, 2H, NH2), 10.53 (s, 1H, NH).


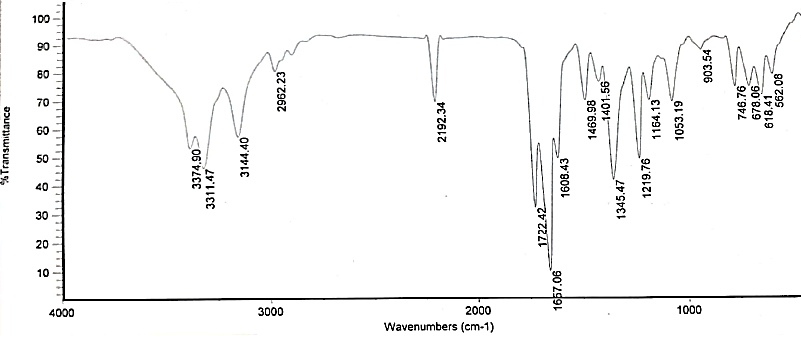


**Figure S1:** FT-IR spectrum of compound **4**a

**Figure S2:** ^1^H NMR spectrum of compound **4**a


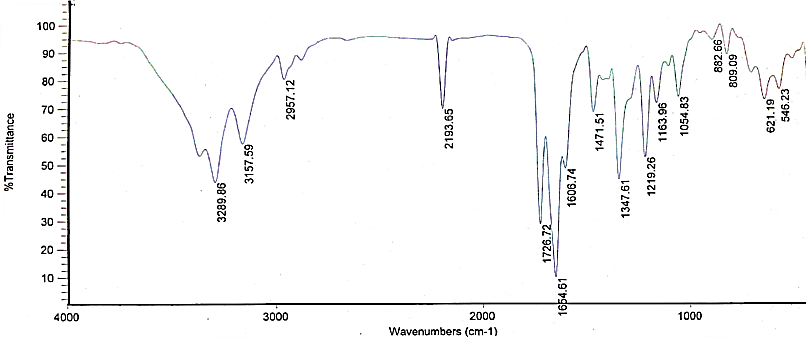


**Figure S3:** FT-IR spectrum of compound **4**b

**Figure S4:** ^1^H NMR spectrum of compound **4**b


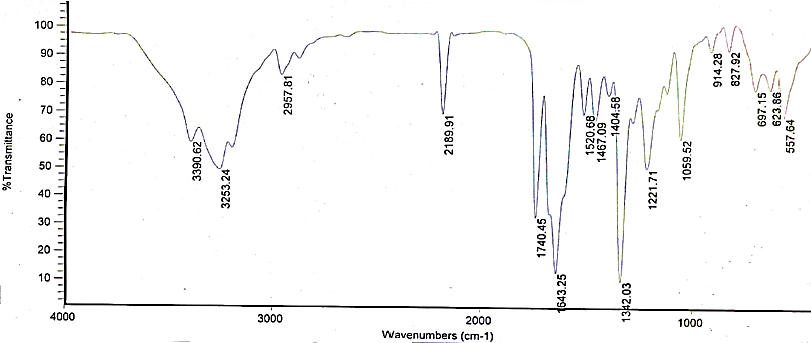


**Figure S5:** FT-IR spectrum of compound **4**c

**Figure S6:** ^1^H NMR spectrum of compound **4**c


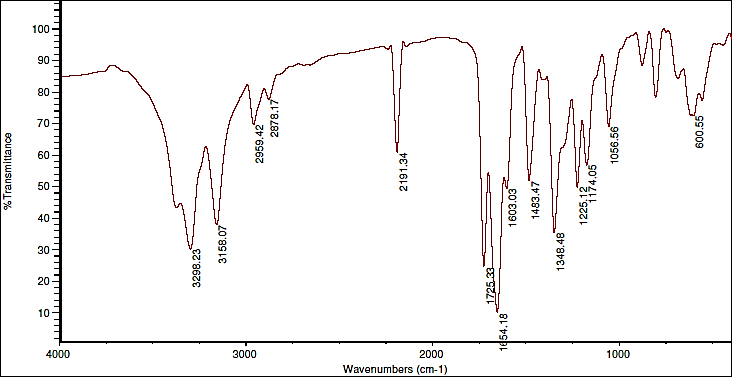


**Figure S7:** FT-IR spectrum of compound **4**d

**Figure S8:** ^1^H NMR spectrum of compound **4**d


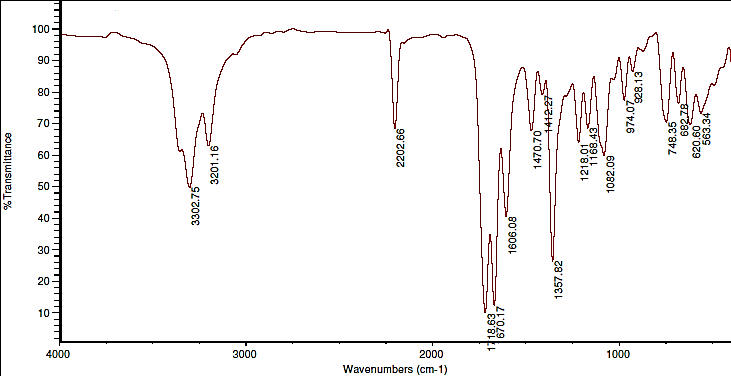


**Figure S9:** FT-IR spectrum of compound **4**e

**Figure S10:** ^1^H NMR spectrum of compound **4**e


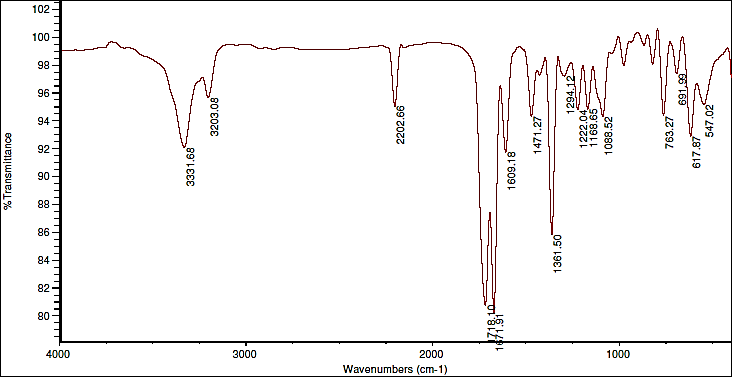


**Figure S11:** FT-IR spectrum of compound **4**f

**Figure S12:** ^1^H NMR spectrum of compound **4**f


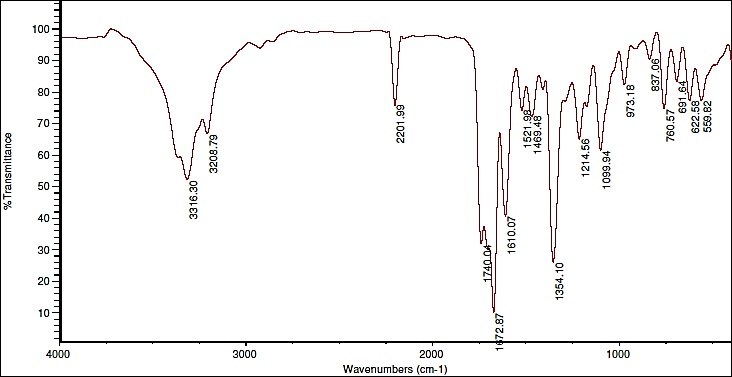


**Figure S13:** FT-IR spectrum of compound **4**g

**Figure S14:** ^1^H NMR spectrum of compound **4**g


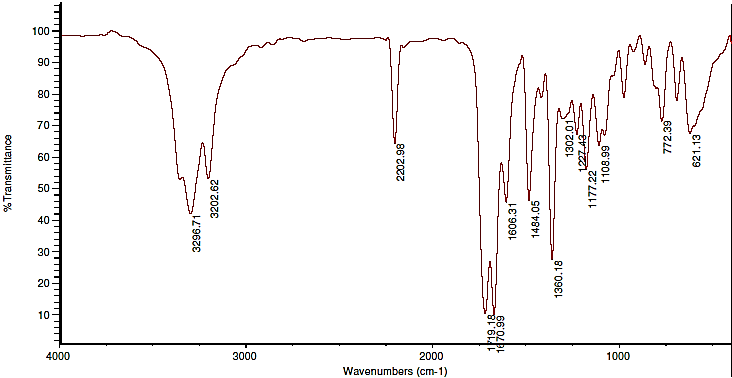


**Figure S15:** FT-IR spectrum of compound **4**h

**Figure S16:** ^1^H NMR spectrum of compound **4**h


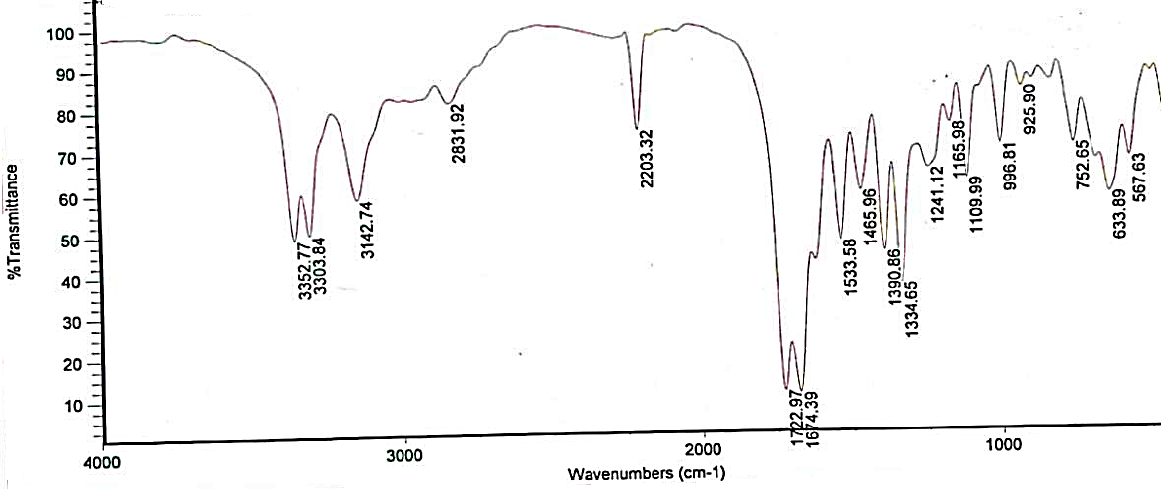


**Figure S17:** FT-IR spectrum of compound **4**i

**Figure S18:** ^1^H NMR spectrum of compound **4**i


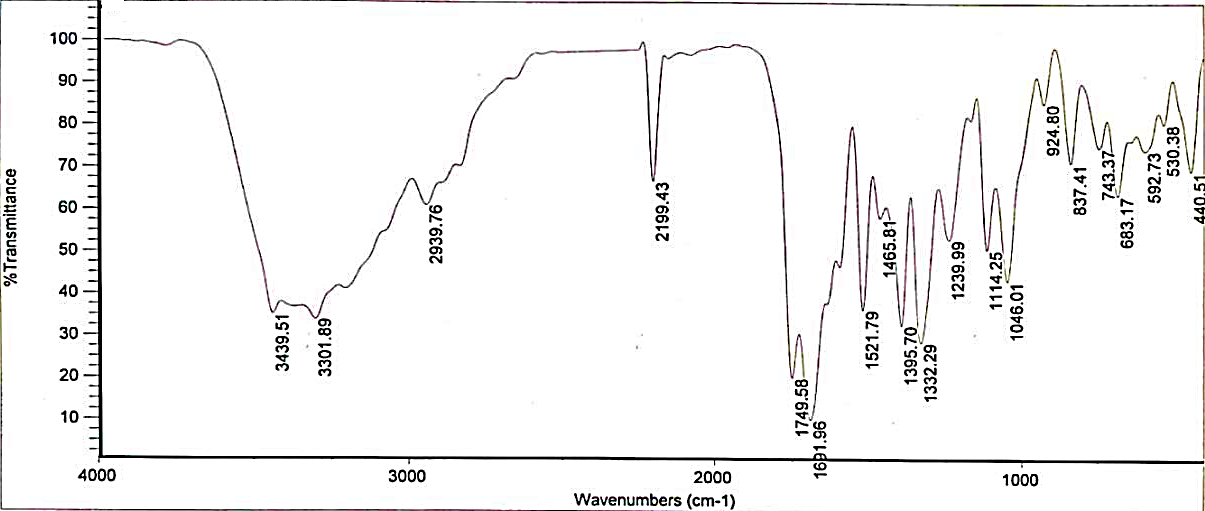


**Figure S19:** FT-IR spectrum of compound **4**j

**Figure S20:** ^1^H NMR spectrum of compound **4**j


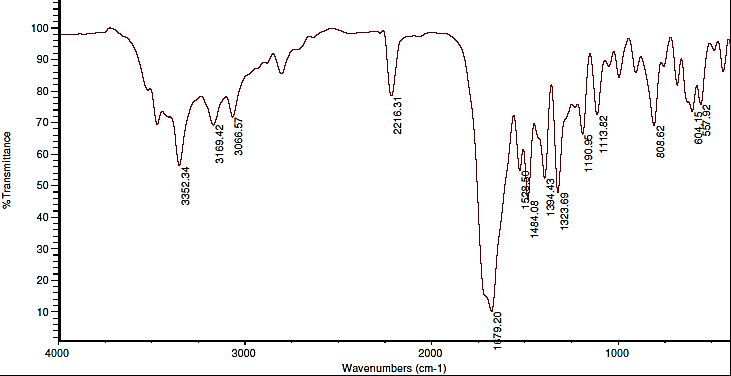


**Figure S21:** FT-IR spectrum of compound **4**k

**Figure S22:** ^1^H NMR spectrum of compound **4**k


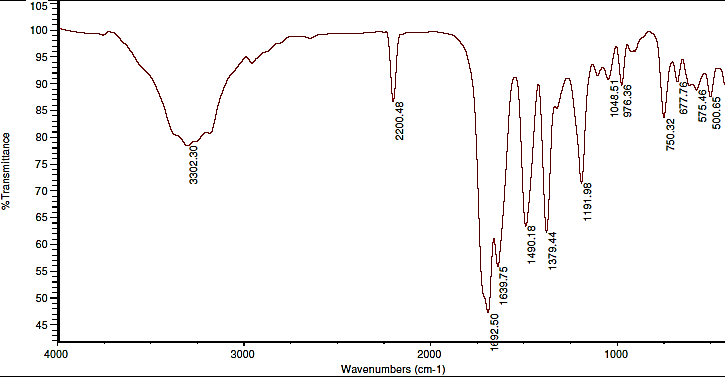


**Figure S23:** FT-IR spectrum of compound **4**l

**Figure S24:** ^1^H NMR spectrum of compound **4**l


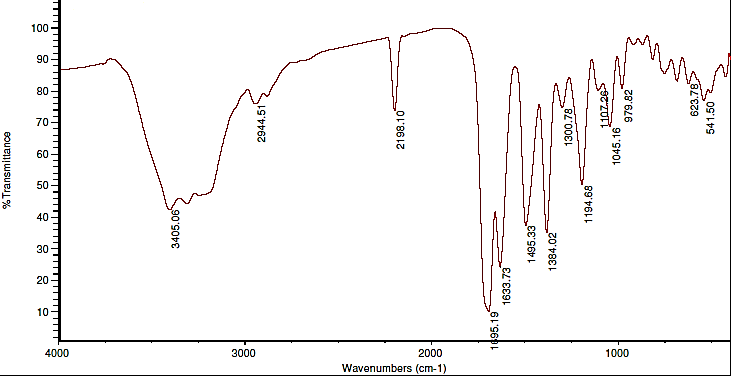


**Figure S25:** FT-IR spectrum of compound **4**m

**Figure S26:** ^1^H NMR spectrum of compound **4**m


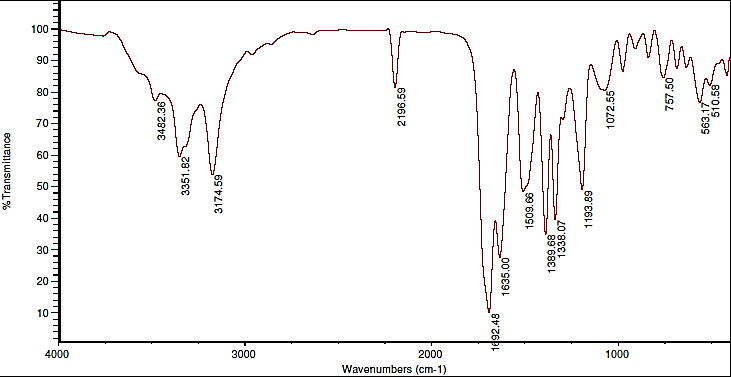


**Figure S27:** FT-IR spectrum of compound **4**n

**Figure S28:** ^1^H NMR spectrum of compound **4**n


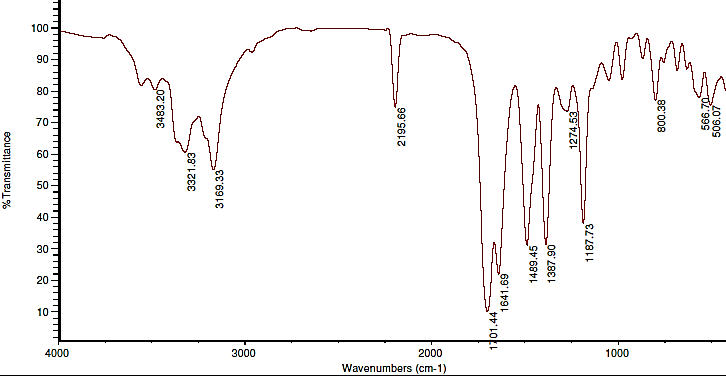


**Figure S29:** FT-IR spectrum of compound **4**o

Figure S30: ^1^H NMR spectrum of compound 3o

**Figure** **S30:** ^1^H NMR spectrum of compound **4**o
